# Amine Switchable Hydrophilic Solvent Vortex-Assisted Homogeneous Liquid–Liquid Microextraction and GC-MS for the Enrichment and Determination of 2, 6-DIPA Additive in Biodegradable Film

**DOI:** 10.3390/molecules29092068

**Published:** 2024-04-30

**Authors:** Kai Cai, Qiang Liu, Yechun Lin, Xingyou Yang, Qi Liu, Wenjie Pan, Weichang Gao

**Affiliations:** 1 Guizhou Academy of Tobacco Science, Guiyang 550081, China; caikai@email.swu.edu.cn (K.C.); linyechun@alu.cau.edu.cn (Y.L.); 2 Qiandongnan Company of Guizhou Province of CNTC, Kaili 556000, China; qiangliu82@163.com; 3 Sichuan Province Company of CNTC, Chengdu 610096, China; yangxingyou@sc.tobacco.gov.cn; 4 National Engineering Laboratory for Crop Efficient Water Use and Disaster Mitigation, Key Laboratory of Prevention and Control of Residual Pollution in Agricultural Film, Ministry of Agriculture and Rural Affairs, Institute of Environment and Sustainable Development in Agriculture, Chinese Academy of Agricultural Sciences, Beijing 100081, China; liuqi@caas.cn

**Keywords:** heating hydrolysis–extraction, amine switchable hydrophilic solvent, vortex-assisted homogeneous liquid–liquid microextraction, 2, 6-diisopropylaniline, PBAT biodegradable films

## Abstract

2, 6-diisopropylaniline (2, 6-DIPA) is a crucial non-intentionally organic additive that allows the assessment of the production processes, formulation qualities, and performance variations in biodegradable mulching film. Moreover, its release into the environment may have certain effects on human health. Hence, this study developed simultaneous heating hydrolysis–extraction and amine switchable hydrophilic solvent vortex-assisted homogeneous liquid–liquid microextraction for the gas chromatography–mass spectrometry analysis of the 2, 6-DIPA additive and its corresponding isocyanates in poly(butylene adipate-co-terephthalate) (PBAT) biodegradable agricultural mulching films. The heating hydrolysis–extraction conditions and factors influencing the efficiency of homogeneous liquid–liquid microextraction, such as the type and volume of amine, homogeneous-phase and phase separation transition pH, and extraction time were investigated and optimized. The optimum heating hydrolysis–extraction conditions were found to be a H_2_SO_4_ concentration of 2.5 M, heating temperature of 87.8 °C, and hydrolysis–extraction time of 3.0 h. As a switchable hydrophilic solvent, dipropylamine does not require a dispersant. Vortex assistance is helpful to speed up the extraction. Under the optimum experimental conditions, this method exhibits a better linearity (0.0144~7.200 μg mL^−1^ with *R* = 0.9986), low limit of detection and quantification (0.0033 μg g^−1^ and 0.0103 μg g^−1^), high extraction recovery (92.5~105.4%), desirable intra- and inter-day precision (relative standard deviation less than 4.1% and 4.7%), and high enrichment factor (90.9). Finally, this method was successfully applied to detect the content of the additive 2, 6-DIPA in PBAT biodegradable agricultural mulching films, thus facilitating production process monitoring or safety assessments.

## 1. Introduction

Poly(butylene adipate-co-terephthalate) (PBAT) is a biodegradable aliphatic–aromatic polyester belonging to petrochemical-based biodegradable plastics. It exhibits good flexibility, elongation at break, high toughness, and heat resistance, along with excellent biodegradability [1]. PBAT is recognized as a sustainable material in the generation of “green material”, considered a promising alternative to traditional film materials, and has been widely used in the production of biodegradable agricultural mulching film [2,3]. During the manufacturing processes, various types of organic additives, such as anti-hydrolysis agents, antioxidants, stabilizers, plasticizers, flame retardants, chain extenders, etc., are added to PBAT biodegradable films to improve their performance [4]. The ecological toxicity of conventional plasticizer additives, such as phthalates, has been extensively reported [5]. Certain additives in PBAT also contain evident toxic units, posing potential emerging environmental pollutants that may impact plant growth, development, soil microbial communities, functions, and human health. Previous studies have emphasized the necessity of considering the ecological impact of additives when evaluating biodegradable materials [6].

**Figure 1:**
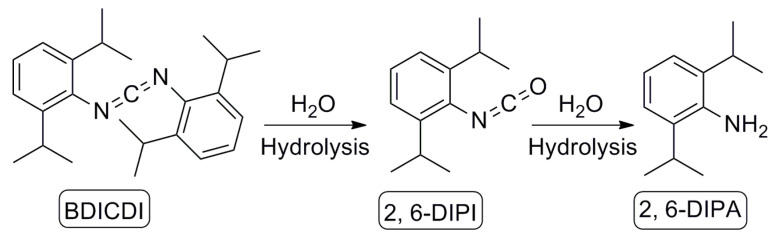
Degradation pathway of BDICDI to 2, 6-DIPI and 2, 6-DIPA in PBAT biodegradable films.

2, 6-diisopropylaniline (2, 6-DIPA) additive as a non-intentionally added substance in PBAT biodegradable films is derived from the degradation of the anti-hydrolysis agent bis (2, 6-diisopropylphenyl) carbodiimide (BDICDI), as illustrated in Figure 1. BDICDI first slowly degrades into the corresponding 2, 6-diisopropylphenyl isocyanate (2, 6-DIPI), ultimately degrading into the chemically stable and quantifiable 2, 6-DIPA. Therefore, as a degradation product, 2, 6-DIPA and 2, 6-DIPI play a crucial indicative role in evaluating the production processes, formulation qualities, and performance variations in biodegradable agricultural mulching films [7]. Additionally, 2, 6-DIPA as a typical aromatic amine suggests potential impacts on human health, which may exhibit significant carcinogenic and mutagenic effects. Exposure to aromatic amines has been linked to a variety of health effects, including bladder, liver, lung, skin cancers, and kidney damage. The mutagenicity can be further activated with the metabolic reaction of *N*-acetylation, oxidation, and conjugation with glucuronic acid. More than one in eight human carcinogens are associated with aromatic amines and their transformation substance [8,9]. Consequently, quantifying 2, 6-DIPA and 2, 6-DIPI additives can facilitate the production process monitoring or safety assessment of PBAT biodegradable agricultural mulching films and related products. However, 2, 6-DIPI can react with a protonated solvent, resulting in the conversion into corresponding byproducts, such as amine, urea, and carbamate [10]. This makes the directly accurate detection of 2, 6-DIPI difficult, and it is necessary to hydrolyze it into 2, 6-DIPA for detecting the total amount of 2, 6-DIPA and 2, 6-DIPI additives.

Extraction, cleanup, and enrichment are key steps in sample preparation for complex matrices. Currently, sample extraction methods for the analysis of additives in plastics include ultrasound-assisted extraction [11], Soxhlet extraction [12], microwave-assisted extraction [13], accelerated solvent extraction [14], and heating extraction [15]. Compared to others, the heating extraction method is relatively simple to operate. It also allows for the hydrolysis of 2, 6-DIPI in an acidic aqueous solution, enabling the simultaneous determination of the total amount of 2, 6-DIPA and 2, 6-DIPI. Sample cleanup and enrichment is an indispensable step for the analysis of additives in plastics. Common methods include conventional solid-phase extraction (SPE) [16], solid-phase microextraction (SPME) [17], dispersive liquid–liquid microextraction (DLLME) [10], and magnetic solid-phase extraction (MSPE) [18]. Switchable hydrophilicity solvent homogeneous liquid–liquid microextraction (SHS-HLLME) has been receiving increasing attention as reflected by a special interest in environmental, food, industrial, pharmaceutical, and biological research for metal ions and organic compounds analysis [19]. SHS has been proposed for the first time by Jessop et al. [20]. SHS is an aqueous solvent capable of reversible switching between homogeneous mixtures and biphasic mixtures. These solvents exhibit a complete miscibility with water in acid, while they become immiscible in base. During the pH switching processes, numerous organic microdroplets can form an emulsion, maximizing the contact area between the two phases and then enhancing the extraction efficiency. These characteristics make it highly suitable for homogeneous microextraction, thus giving rise to SHS-HLLME. This technique offers many advantages such as greenness, ease of applicability, cost-effectiveness, and rapidness of separation, allowing for rapid and simple sample cleanup and enrichment methods [21]. However, SHS-HLLME has not previously been used in polymer matrices.

2, 6-DIPA belongs to the aromatic amine compounds. However, no relevant literature has been reported regarding its detection. The methods commonly used for determining amine with a similar structure include gas chromatography (GC) [22], high-performance liquid chromatography (HPLC) [23], capillary electrophoresis (CE) [24], and ion chromatography [25]. Derivatization with GC-MS has been recognized as the method of choice for aromatic amine, due to its superiority in selectivity and sensitivity [26]. However, the nitrogen atom is shielded by two isopropyl groups in 2, 6-DIPA, and its alkalinity and polarity are significantly reduced. This characteristic makes it suitable for direct injection gas chromatographic analysis with good gas chromatographic properties.

This study aims to address the need for a reliable and efficient analytical method for quantifying 2, 6-DIPA and related additives, which are commonly used in agricultural film production. Hence, amine switchable hydrophilic solvent vortex-assisted homogeneous liquid-phase microextraction (SHS-VAHLLME) and GC-MS were established for the enrichment and determination of 2, 6-DIPA and related 2, 6-DIPI additive in PBAT biodegradable agricultural mulching film. This study mainly focuses on (1) optimizing the heating hydrolysis–extraction conditions through response surface methodology; (2) testing whether SHS-VAHLLME can enrich 2, 6-DIPA in complex plastic matrices; and (3) validating and applying the developed method to monitor the content of 2, 6-DIPA additive in biodegradable agricultural mulching film. To the best of our knowledge, this is the first work describing heating hydrolysis–extraction and amine SHS-VAHLLME for the extraction and enrichment of 2, 6-DIPA additive from polymer matrices.

## 2. Results and Discussion

### 2.1. Selection of Internal Standards

The selection of the appropriate internal standard is important for obtaining accurate results. A good internal standard should be well separated from the target analytes, which have the same or similar physical and chemical properties, be easily obtained, be stable under experimental conditions, and not exist in the samples [27]. 2, 6-diethylaniline (DEA), 2, 4, 6-tri-tert-butyl aniline (TBA), and 2, 4, 6-trimethyl aniline (TMA) were selected for screening. The pK_a_ values of these three compounds are 4.13, 3.30, and 5.38, respectively, while the pK_a_ of 2, 6-DIPA is 4.25. TMA has strong alkalinity, which results in a strong tailing peak and matrix effect during quantification, while TBA has weak alkalinity, which is prone to loss under heating with a high temperature. Therefore, DEA was chosen as an internal standard for recovery correction during sample heating hydrolysis–extraction and TBA as a surrogate for instrument calibration after sample preparation.

### 2.2. Optimization of Heating Hydrolysis–Extraction Procedures

#### 2.2.1. Effect of Type of Hydrolysis Solvent

**Figure 2:**
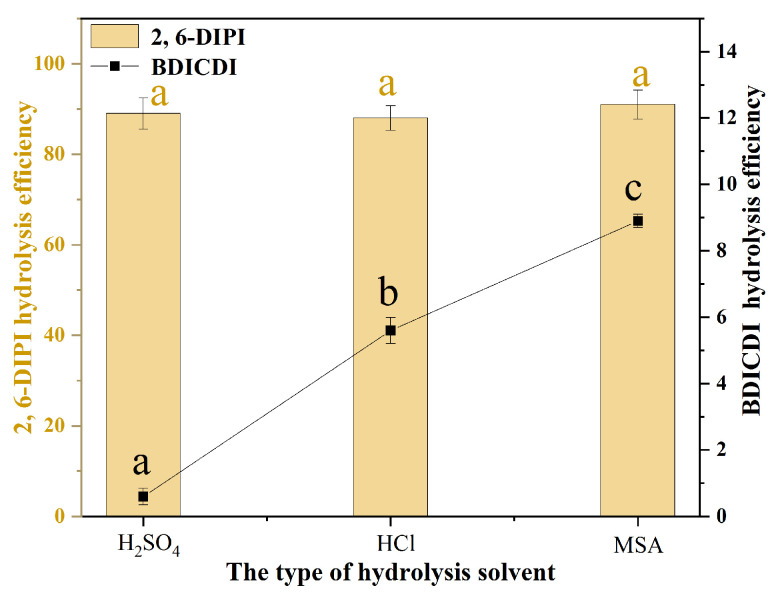
The optimization of hydrolysis efficiencies of 2, 6-DIPI and BDICDI with different acid solutions in H_2_SO_4_, HCl, and MSA. Different superscript letters denote significant differences (*p* < 0.05).

Both free 2, 6-DIPA and 2, 6-DIPI exist in the PBAT biodegradable agricultural mulching film, and it is necessary to hydrolyze 2, 6-DIPI into 2, 6-DIPA with an acid aqueous solution for detecting the total amount [7]. In this experiment, 3 mol L^−1^ H_2_SO_4_, 6 mol L^−1^ HCl, and 6 mol L^−1^ methylsulfonic acid (MSA) were used for the hydrolysis and extraction of 2, 6-DIPI, due to the differences between monoacid and diacid. In addition, to evaluate their hydrolysis efficiencies for the BDICDI parent compound, 100 μL of a high-concentration BDICDI standard solution (10.300 mg mL^−1^) was heated at 90 °C for 3 h for hydrolysis. The hydrolysis efficiencies of 2, 6-DIPI and BDICDI with different acids are shown in Figure 2. The results revealed that the three acids have no significant difference in the hydrolysis efficiency of 2, 6-DIPI. However, HCl and MSA had significantly higher hydrolysis efficiencies for BDICDI than H_2_SO_4_. H_2_SO_4_ exhibits the lowest hydrolysis efficiency with 0.65%. The reason is that the high permeability of HCl and MSA for a polymer raw material leads to better hydrolysis efficiency. An interaction force between hydrophobic methyl and water molecules caused a strong repulsion to push MSA into the polymer [28], whereas HCl has a high diffusion coefficient in a closed reaction vessel [29]. To reduce the interference from BDICDI, H_2_SO_4_ was chosen for the heating hydrolysis–extraction of 2, 6-DIPA and 2, 6-DIPI from PBAT biodegradable agricultural mulching films because BDICDI was widely used as an efficient stabilizer suppressing the hydrolytic scission of ester bonds in PBAT materials [30]. Under the conditions of HCl and MSA, the hydrolysis efficiency of BDICDI was higher, leading to an overestimation of 2, 6-DIPA concentration.

#### 2.2.2. Optimization of 2, 6-DIPI Hydrolysis Efficiency with BBD

An advantage of BBD is that it is more efficient, does not have axial points, and may be experimentally more convenient and less expensive [31]. The uncoded variable factors and response factor are listed in Table 1. The BBD included 17 experiments with five center points. The 2, 6-DIPI extraction recovery was used as a response factor. To evaluate the fit between the model and the experimental results, BBD was evaluated at a 5% significance level and validated using analysis of variance (ANOVA), as shown in Table 1. The results indicated that the model for 2, 6-DIPA hydrolysis efficiency is highly significant, with an F-value of 50.59 and a *p*-value < 0.0001. The coefficient of determination (*R*^2^) is 0.985. A lack-of-fit *p*-value of 0.1941, greater than 0.05, suggests no significant correlation with pure error. Moreover, the coefficient of variation (CV) was used to measure the signal-to-noise ratio. A ratio greater than 4 is desirable. The ratio of 22.71 indicates an adequate signal. These statistical parameters demonstrate the reliability of the model.

**Figure 3:**
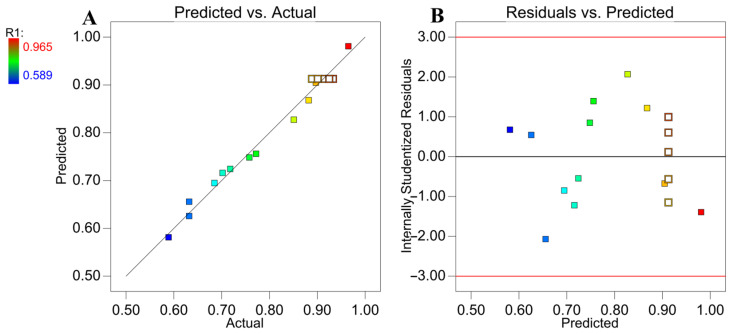
Diagnostic plots for BBD model adequacy: (**A**) predicted values versus actual values; (**B**) internally studentized residuals versus predicted values, the red lines (±3) are limit value.

To predict the optimum value with this model, the variables were set according to our specific requirements. The concentration of H_2_SO_4_ (A) had no significant impact on the hydrolysis efficiency. To reduce the hydrolysis risk of BDICDI, a level of 0 at 2.5 M was selected. Heating temperature (B) and hydrolysis–extraction time (C) were further screened for optimization with Design-Expert. The variables B and C were set as “in range” from the lower −1 to upper +1 and then the objective for the extraction recovery of 2, 6-DIPI was set to maximize, as the maximum value was the target. The highest extraction recovery (98.8%) was obtained with a heating temperature of 87.8 °C and hydrolysis–extraction time of 3.0 h. The above results indicated that 2, 6-DIPI is readily hydrolyzable under acidic conditions; however, due to significant steric hindrance, a longer reaction time is required to achieve optimal hydrolysis efficiency [32]. A verification experiment was carried out under the optimal conditions. The experimental value was 97.7% ± 1.15%, showing no significant difference from the optimal value. These results showed that this model is sufficient to reflect the anticipated optimization. In addition, the correlation analysis between the predicted values and the actual values, as shown in Figure 3A, where points are evenly distributed on both sides of y = x, further proved a satisfactory model’s reliability [33]. Figure 3B shows the relationship between the internally studentized residuals versus the predicted. The limit of the internally studentized residuals was found to be ±3 by the distribution of points scattered randomly around the boundary [34]. These diagnostic plots survey the reliability and adequacy of the models, and a constant variance was seen through the response range. Additionally, the hydrolysis efficiency of the high-concentration BDICDI standard solution was only 0.52%, indicating that these conditions are suitable for the hydrolysis and extraction of 2, 6-DIPI in biodegradable agricultural mulching films.

### 2.3. Optimization of Amine SHS-VAHLLME Conditions

In the amine SHS-VAHLLME processes of 2, 6-DIPA, different factors such as the type and volume of amine, homogeneous-phase and phase separation transition pH, and extraction time influence the extraction efficiency. To achieve the optimal conditions, one-factor-at-a-time was used for optimization. The extraction efficiency was evaluated using R × V_org_/R_surrogate_, where R and R_surrogate_ are the peak area of the 2, 6-DIPA and TBA, and V_org_ is the volume of the organic phase.

#### 2.3.1. Effect of Type of Amines

**Figure 4:**
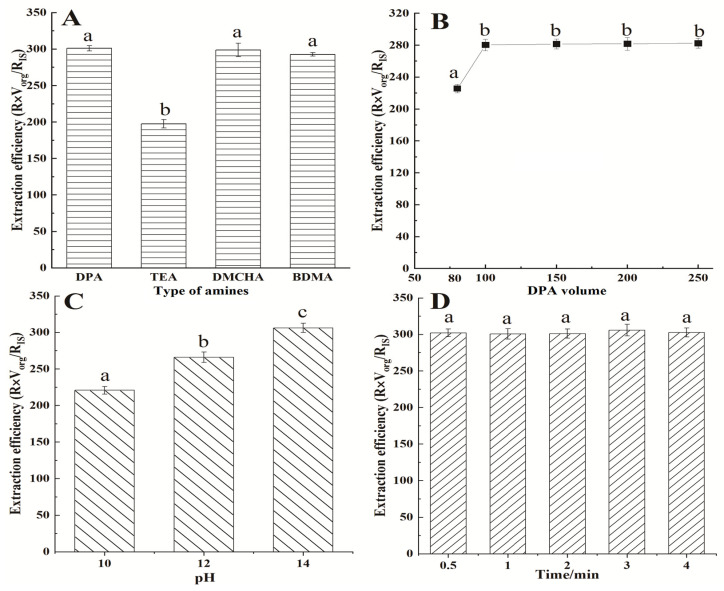
Effect of type of amines (**A**), DPA volume (**B**), transition pH (**C**), and extraction time (**D**) on the extraction efficiency of 2, 6-DIPA. Different superscript letters denote significant differences (*p* < 0.05).

The amine switchable hydrophilic solvent needs to meet the following two requirements: Firstly, the octanol/water partition coefficient (log K_ow_) must fall between 1.2 and 2.5. Secondly, the strength of the conjugate base (pK_a_) should be close to or higher than 9.5 [35]. Based on the above criteria, TEA (log K_ow_ = 1.47, pK_a_ = 10.68), DPA (logK_ow_ = 1.64, pK_a_ = 11.05), DMCHA (log K_ow_ = 2.04, pK_a_ = 10.48), and BDMA (log K_ow_ = 1.86, pK_a_ = 9.03) were selected. An amount of 200 μL of triethylamine (TEA), dipropylamine (DPA), *N*, *N*-dimethylcyclohexylamine (DMCHA), and benzyldimethylamine (BDMA) was added for SHS-VAHLLME. The results showed that all amine switchable hydrophilic solvents could achieve phase separation. As seen in Figure 4A, TEA had the lowest volume of the organic phase and relatively lower efficiency, indicating higher water solubility. Other solvents had a similar extraction efficiency. However, BDMA showed multiple interference peaks and DMCHA showed larger errors in the repeated sample analysis. Further, DPA is a biodegradable, commercially available, and inexpensive amine, and can have improved switching speeds compared to tertiary amines. They also biodegrade more readily than tertiary amines. Considering the comprehensive effect, DPA was selected in the subsequent studies.

#### 2.3.2. Effect of DPA Volume

The sample volume was kept constant at 4 mL and the effect of the DPA volume was investigated in the range of 50–250 µL. It was observed that 50 μL of DPA could not form an effective organic layer. This behavior was also observed in previous studies [36]. Due to the use of the μ-pipette device, lower amounts of DPA can be effectively sampled at the upper organic phase [37]. As shown in Figure 4B, the extraction efficiency increases with the increase in the DPA volume. When the amount reached 100 μL, the extraction efficiency remained relatively stable. Due to the dilution effect, the enrichment factor is reduced with the increasing DPA volume. Therefore, 100 μL DPA was chosen as the optimum volume.

#### 2.3.3. Effect of Transition pH

Some of the literature has shown that a homogeneous phase can be formed with an acid solution instead of commonly used dry ice CO_2_ [38]. In this experiment, the hydrolysis–extraction solution was H_2_SO_4_, and 1.5 M H_2_SO_4_ solution was sufficient to form a large amount of homogeneous solution with DPA microdroplets. To reduce the steps, no adjustment of the pH was made to form a homogeneous phase. Then, it is necessary to convert the hydrophilicity into hydrophobicity in an alkaline environment, achieving DPA phase separation and completing the extraction process. Furthermore, the pH of the sample solution is also a critical factor for target compounds with an acidic or basic functional group [39]. The distribution coefficient and extraction efficiency were drastically changed upon the switching between ionic and neutral forms. The pH was adjusted to 8, 10, 12, and 14 with 12 mol L^−1^ NaOH for phase separation, respectively. As seen from Figure 4C, when the pH of the system was 8, phase separation did not occur. As the pH increased, the volume of DPA gradually increased. When the pH was 14, the extraction efficiency of 2, 6-DIPA reached its maximum. Therefore, pH = 14 was chosen as the optimum phase separation condition.

#### 2.3.4. Effect of Salt Addition and Extraction Time

The presence of NaCl may affect the extraction efficiency through the salting-out effect [40]. Thus, various concentrations of sodium chloride (0–10%, *w*/*v*) were studied. An amount of 5–10% NaCl was not considered due to its adverse effect on the phase separation of DPA. The results showed that the addition of NaCl has no significant effect on the extraction efficiency of 2, 6-DIPA. In the amine SHS-VAHLLME extraction processes, the extraction time is defined as the time from the addition of NaOH for forming micro-droplets to before phase separation. Different extraction times of 0.5, 1, 2, 3, and 4 min were investigated. As shown in Figure 4D, the amine SHS-VAHLLME extraction process was completed in a short time with the vortex method, and the extraction time has no significant influence on the extraction efficiency. This phenomenon is attributed to the large contact surface area between the extraction solvent and sample solution [41]. According to the obtained results, no additional salt was used, and 0.5 min extraction times were selected for further experiments.

### 2.4. Method Validation and Evaluation

**Figure 5:**
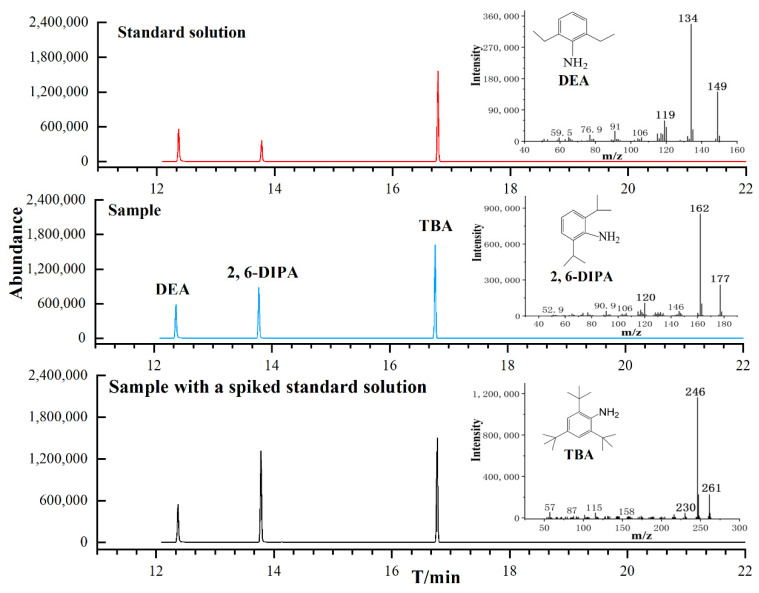
Typical GC-MS-SIM chromatogram and mass spectrum of each compound in standard solution, sample (PBAT biodegradable agricultural mulching film), and sample with a spiked standard solution. The bold numbers of mass spectrum were quantitative ions for DEA (*m*/*z* 134), 2, 6-DIPA (*m*/*z* 162), and TBA (*m*/*z* 246) and qualitative ions for DEA (*m*/*z* 149, 119), 2, 6-DIPA (*m*/*z* 177, 120), and TBA (*m*/*z* 261, 230).

Under optimal conditions, the linearity, limits of detection (LOD), limits of quantification (LOQ), extraction recovery, intra- and inter-day precise, and enrichment factor (EF) of 2, 6-DIPA were evaluated to validate the proposed method. For linearity, the internal calibration curve (y = ax + b) was constructed using weighted (1/X) least-squares linear regression models, by plotting the peak area ratio (y) using the internal standard (10 μL with concentration 1.080 mg mL^−1^ DEA) versus the gradient concentration (x). To check the linearity, the correlation coefficient (*R*) was applied. The regression equation is y = 0.2377x − 0.0049 in Figure S1, with an *R* = 0.9986 in the range of 0.0144~7.200 μg mL^−1^. The LOD and LOQ (in μg g^−1^) were calculated as 3 and 10 times the signal-to-noise ratio (S/N) of the lowest standard solution concentration of the calibration curve, considering the sample weight, dilution ratio, and recovery. The LOD and LOQ of 2, 6-DIPA were 0.0033 μg g^−1^ and 0.0103 μg g^−1^. These results showed that the developed method provides a wide range of linearity and high sensitivity. The typical GC-MS-SIM chromatogram and mass spectrum of each compound in the standard solution, sample, and sample with a spiked standard solution are shown in Figure 5.

The extraction recovery of 2, 6-DIPA was tested by spiking with approximately half of the initial concentration (low spiking level) and the initial concentration (high spiking level) in PBAT biodegradable agricultural mulching film. Due to the significant variation in 2, 6-DIPA content among the samples, validation was conducted separately using samples with low (131.1 µg g^−1^) and high (2872.4 µg g^−1^) concentrations. The spiked sample was incubated overnight at 4°C and then extracted. The extraction recovery (R, %) was calculated using the equation, R% = {[(Concentration of the spiked sample − Concentration of the unspiked sample)/Spiked concentration] × 100}. The intra- and inter-day precision was expressed as the relative standard deviation, which was calculated by analyzing five independent samples within a single day and on three consecutive days, respectively. Stabilities were tested by relative differences (RDs) between the analyte concentration at t = 0 and the end of the storage period (t = 7 d). As shown in Table 2, the extraction recovery was 92.5–105.4% and 96.7–105.0% in the low- and high-concentration samples, respectively. The intra- and inter-day precision was less than 4.1% and 4.7%. Stability RDs were between −1.3% and 2.4%. The EF was found to be 90.9, which was calculated as the ratio of the final concentration of the analyte in the DPA and sample solution. All these parameters meet strict qualitative and quantitative requirements.

### 2.5. Sample Analysis in PBAT Biodegradable Agricultural Mulching Films

Ten PBAT biodegradable agricultural mulching films with different origins were collected, and the total amount of 2, 6-DIPA and 2, 6-DIPI additives was analyzed using the developed method, as shown in Table 3 and Figure S2. All samples contained 2, 6-DIPA or 2, 6-DIPI additives with levels exceeding 85.1 µg g^−1^. 2, 6-DIPA or 2, 6-DIPI is mainly derived from the degradation of the anti-hydrolysis agent BDICDI [7]. These results revealed that most PBAT biodegradable agricultural mulching films are added with BDICDI during the production process. BDICDI can remove water and acid from composite materials and then inhibit the cleavage of ester bonds. These functions can effectively prevent the self-catalytic degradation of PBAT and improve the stability of the material [42]. The high content of 2, 6-DIPA or 2, 6-DIPI (reaching the level of mg g^−1^) indicated the production processes need to adjust to reduce the degradation of BDICDI during production. Additionally, considering the extensive adaptability of SHS-VAHLLME to diverse switchable hydrophilic solvents (amine and fatty acid-based), its application could be potentially extended to the quantitative analysis of organic and inorganic additives in various polymer matrices [43].

## 3. Materials and Method

### 3.1. Reagents, Standard Solutions, and Samples

The standards of BDICDI, 2, 6-DIPI, 2, 6-DIPA, internal standards (ISs) of DEA, TBA, TMA, and amine switchable hydrophilic solvent of TEA, DPA, DMCHA, and BDMA were obtained from Aladdin Industrial Co., Ltd. (Shanghai, China) with purities ≥ 98%. The MSA, HCl, H_2_SO_4_, and other reagents were procured from Sinopharm Chemical Reagent Co., Ltd. (Beijing, China). The standard stock solutions of BDICDI (10.30 mg L^−1^), 2, 6-DIPI (1.02 mg L^−1^), and 2, 6-DIPA (10.80 mg L^−1^) were prepared with acetone and stored in brown bottles at 4 °C. Standard working solutions were prepared fresh immediately before use by diluting with acetone. The IS solutions of DEA (1.080 mg L^−1^), TBA (2.808 mg L^−1^), and TMA (0.468 mg L^−1^) were also prepared with acetonitrile and stored in brown bottles at 4 °C.

To compare the differences in 2, 6-DIPA in PBAT biodegradable agricultural mulching films from different manufacturers, ten PBAT samples (PBAT-1, PBAT-2, PBAT-3, PBAT-4, PBAT-5, PBAT-6, PBAT-7, PBAT-8, PBAT-9, and PBAT-10), which are widely used in agricultural production, were selected. Three repetitive samples were taken and then cut into fragments of approximately 2 mm × 2 mm. These samples were stored in glass bottles for further use.

### 3.2. Apparatus

GC-MS analyses were carried out on an Agilent 7890A-5975C with CTC PAL auto-injection (Agilent Technologies, Palo Alto, CA, USA). System control and data acquisition were accomplished with Chemstation E02.02 software. Chromatographic separations were performed on a fused silica capillary column DB-5 (30 m × 0.25 mm i.d. × 0.25 μm film thickness) using helium (purity 99.999%) as carrier gas at a constant flow rate of 1 mL min^−1^. The column temperature program was as follows: initial temperature 40 °C, hold for 1 min; then ramped at 10 °C min^−1^ to 230 °C, hold for 1 min; ramped at 15 °C min^−1^ to 280 °C, hold for 0 min. The total run time was 24.33 min. A split injection mode with a split ratio of 20:1 was selected for the injection volume of 1 µL. The injector was kept at 280 °C. The temperature of the ion source and quadrupole were maintained at 230 °C and 150 °C, respectively. The electron impact ionization energy was 70 eV, and the transfer line temperature was 280 °C. The solvent delay was set to 12 min. Mass spectral data were acquired in full scan with mass-to-charge (*m*/*z*) ranging from 45 to 500 amu. Quantitative analysis was performed in the selected ion monitoring (SIM) mode and SIM quantitative ions for DEA, 2, 6-DIPA, and TBA were *m*/*z* 134, 162, and 246. The qualitative ions were *m*/*z* 149, 119 for DEA, 177, 120 for 2, 6-DIPA, and 261, 230 for TBA.

### 3.3. Heating Hydrolysis–Extraction of 2, 6-DIPI and 2, 6-DIPA

A 50 mg aliquot of PBAT biodegradable agricultural mulching film was weighed into a 20 mL headspace vial, and then 10 μL of IS solution DEA with a concentration of 1.080 mg mL^−1^ was added. An amount of 3 mL of 2.5 mol L^−1^ H_2_SO_4_ solution was added and heated with a constant temperature metal bath at 87.8 °C for 3 h to hydrolyze 2, 6-DIPI and then extract 2, 6-DIPA. After cooling to room temperature, the extract solution was filtered through a 0.22 μm nylon membrane into a 10 mL glass centrifuge tube for later use.

### 3.4. Amine SHS-VAHLLME Procedures

**Figure 6:**
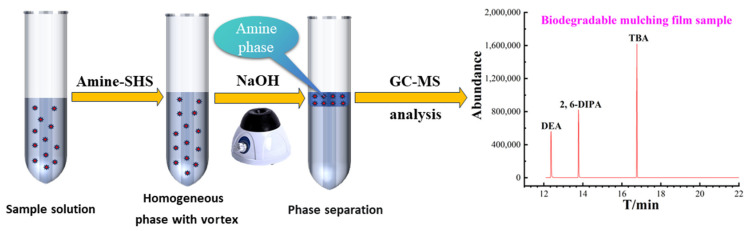
A schematic diagram of the proposed amine SHS-VAHLLME method.

A schematic diagram of the amine SHS-VAHLLME based on the sample cleanup and enrichment method is shown in Figure 6. In a typical procedure, 2 mL of the sample extract solution was transferred to a 10 mL centrifuge tube and then 2 mL of ultrapure water was added. Then, 100 μL of DPA was added to the sample for 10 s of vortex mixing until a homogenous system was observed. The tube was placed in an ice bath for 2 min and then 12 mol L^−1^ NaOH was added to adjust the pH to 14 (because a high concentration of NaOH would cause the solution to heat up, resulting in the volatilization of DPA, the ice bath should be operated for keeping relatively constant DPA volume). At this step, a cloudy solution was immediately formed, and the extraction was performed with vigorous vortex mixing for 30 s. Then, the phase separation of the DPA was obtained after standing for about 3 min. The DPA phase was collected using a μ-pipette device from the upper organic layer into a 100 μL polypropylene insert with polymer feet. The μ-pipette device consisted of coupling a 1 mL Pasteur pipette tip with a 100 μL tip. The final volume of the organic layer was about 60 μL. Finally, the surrogate of 2 μL 2.808 mg mL^−1^ TBA was added for calibration of the instrument and then 1 μL solution was analyzed with GC-MS.

### 3.5. Response Surface Methodology Analysis with BBD

A multivariate strategy based on the second-order response surface methodology with a three-factor, three-level BBD was employed to improve the hydrolysis efficiency of 2, 6-DIPI. The concentration of H_2_SO_4_ (A), heating temperature (B), and hydrolysis–extraction time (C) were selected as independent factors and studied at three levels based on single-factor pre-experiments. The experimental parameter ranges and variable levels are presented in Table 4. The variable levels were set as low (−1), middle (0), and high (+1) values and correspond with the parameter ranges of A (−1 = 1.0, 0 = 2.5, +1 = 4.0), B (−1 = 50, 0 = 80, +1 = 110), and C (−1 = 0.5, 0 = 1.75, +1 = 3). The optimization was performed using 50 mg aliquot of PBAT biodegradable agricultural mulching film with the addition of 100 μL of 2, 6-DIPI standard stock solutions, and then H_2_SO_4_ solution was added and heated for hydrolysis of 2, 6-DIPI. The extraction recovery of 2, 6-DIPI was used as the optimization criterion for evaluating hydrolysis efficiency.

### 3.6. Statistical Analysis

The conditions of 2, 6-DIPI hydrolysis efficiency were optimized with a three-factor, three-level BBD response surface methodology. ANOVA of BBD was conducted with the Design-Expert version 8.0.6 software (Stat-Ease Inc., Minneapolis, MN, USA) to justify the model adequacy. To assess how well the proposed model fit, parameters such as model *p*-value, lack of fit, CV, and *R*^2^ were used. The model *p*-value and CV less than 0.05 and 10%, lack of fit, and *R*^2^ greater than 0.05 and 0.90 showed a reliable model [44]. The Student’s *t*-test with Fisher’s least significant difference (LSD) test was applied for optimization of the conditions of amine SHS-VAHLLME and statistical comparison of 2, 6-DIPA concentration. Differences were considered significant using SPSS 16.0 software (SPSS Inc., Chicago, IL, USA) when *p* < 0.05. All diagrams were drawn using Origin 8.0 Software (Origin Lab Corp., Northampton, MA, USA) and ChemBioDraw Ultra 7.0 (Cambridgesoft.com, Cambridge, MA, USA).

## 4. Conclusions

In this study, heating hydrolysis combined with amine SHS-VAHLLME has been introduced as a novel method for the extraction, cleanup, and enrichment of the total amount of 2, 6-DIPA and 2, 6-DIPI additives, and then analyzed with GC-MS. The optimal heating hydrolysis–extraction conditions were obtained with BBD response surface methodology. The switchable change in the hydrophilicity of DPA as the extractant was performed using HCl and NaOH. The vortex was considered to accelerate the formation of fine droplets and reduce the equilibration time. The method was fully validated with a wide linear range, high extraction recoveries, and good precision. Additionally, DPA SHS-VAHLLME had ease of operation, high enrichment factors, and low toxicity. The developed method provides a feasible approach for the quantification of the total amount of 2, 6-DIPA and 2, 6-DIPI additives in different origin PBAT biodegradable agricultural mulching films. Despite this method having certain advantages, it also suffers from drawbacks such as being relatively labor-consuming with many steps for the likelihood of error. Additionally, there is no perfect SHS that can achieve fully green analytical chemistry. Therefore, online coupling of these methods with automation remains a significant challenge that needs to be taken into account in future studies. More SHS types also need to be investigated for more environmentally friendly alternatives.

## Figures and Tables

**Table 1:** Box–Behnken design: uncoded variables, and response factor with 2, 6-DIPI extraction recovery.

	Uncoded Variable Factors			Response Factor
Nos.	A	B	C	Extraction Recovery
1	0.5	50	1.75	63.2%
2	4	110	1.75	85.1%
3	0.5	80	3	88.2%
4	0.5	80	0.5	77.2%
5	2.25	110	0.5	68.5%
6	2.25	80	1.75	90.1%
7	0.5	110	1.75	71.8%
8	2.25	50	0.5	58.9%
9	2.25	110	3	89.7%
10	4	50	1.75	63.2%
11	2.25	80	1.75	92.5%
12	2.25	80	1.75	93.3%
13	4	80	3	96.5%
14	2.25	50	3	75.8%
15	4	80	0.5	70.2%
16	2.25	80	1.75	88.9%
17	2.25	80	1.75	91.5%
ANOVA	Model F and *p*-value			50.59 and <0.0001
	Variable importance A			0.059
	Variable importance B			<0.0001
	Variable importance C			<0.0001
	Lack-of-fit *p*-value			0.1941
	Coefficient of variation			22.71
	*R* ^2^			0.985

**Table 2:** Recovery and precision of 2, 6-DIPA with the proposed method in PBAT biodegradable agricultural mulching films.

	Spiked/ µg g^−1^	Recovery of Repeated Samples/%					Mean Recovery/%	Inter-Day Precision /RSD%	Intra-Day Precision /RSD%	Stability /%
		1	2	3	5	6				
Low-concentration samples	64.8	94.5	105.4	103.6	97.7	104.2	101.1	4.7	4.1	2.4
	129.6	95.0	92.5	99.2	95.7	98.3	96.1	2.8	2.6	−1.3
High-concentration samples	1411.2	98.4	97.4	103.8	105.0	98.3	100.6	3.5	3.2	2.2
	2822.4	96.7	97.0	96.8	102.3	97.0	98.0	2.5	2.3	1.8

**Table 3:** The content of 2, 6-DIPA or 2, 6-DIPI in ten PBAT biodegradable agricultural mulching films.

Samples	2, 6-DIPA Content/µg g^−1^	Samples	2, 6-DIPA Content/µg g^−1^
PBAT-1	2875.5 ± 276.5 ^1^	PBAT-6	144.9 ± 16.4
PBAT-2	131.9 ± 16.8	PBAT-7	2729.5 ± 255.9
PBAT-3	85.1 ± 10.6	PBAT-8	153.2 ± 12.3
PBAT-4	103.3 ± 8.8	PBAT-9	108.9 ± 11.7
PBAT-5	99.2 ± 15.5	PBAT-10	105.3 ± 22.3

^1^ Data presented as mean ± SD for three repetitive samples.

**Table 4:** Experimental parameter ranges and variable levels of BBD.

Levels	Variable Factors		
	H_2_SO_4_ Concentration (A)/M	Heating Temperature (B)/°C	Hydrolysis–Extraction Time (C)/h
Low (−1)	1.0	50	0.5
Middle (0)	2.5	80	1.75
High (1)	4.0	110	3

## Data Availability

The datasets used during the current study are available from the corresponding author on reasonable request.

## Supplementary Materials

The following supporting information can be downloaded at: https://www.mdpi.com/article/10.3390/molecules29092068/s1, Figure S1: The internal calibration curve (y = ax + b) of 2, 6-DIPA using weighted (1/X) least-squares linear regression models (A); The corresponding each data of internal calibration curve (B); Figure S2: Typical raw GC-MS-SIM chromatogram of high (A: PBAT-1) and low (B: PBAT-2) concentration samples (12.37 min, DEA; 13.77, 2, 6-DIPA; 16.77, TBA).
