# Supplementary material for: Amine Switchable Hydrophilic Solvent Vortex-Assisted Homogeneous Liquid–Liquid Microextraction and GC-MS for the Enrichment and Determination of 2, 6-DIPA Additive in Biodegradable Film
Source: Molecules. 2024 Apr 30;29(9):2068. doi: 10.3390/molecules29092068 (PMC11085926; doi:10.3390/molecules29092068)

Figure S1. The internal calibration curve (y=ax+b) of 2, 6-DIPA using weighted (1/X) least-squares linear regression models (A); The corresponding each data of internal calibration curve (B)

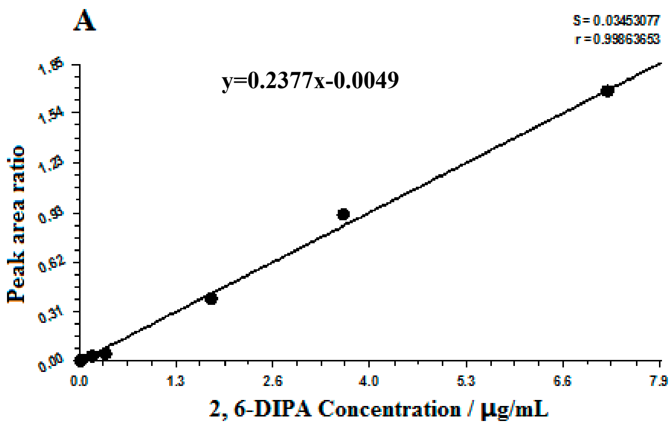

B

2, 6-DIPA Concentration	Peak area ratio
0.0144	0.006549
0.0288	0.007168
0.036	0.011189
0.18	0.033768
0.36	0.05208
1.8	0.394047
3.6	0.915058
7.2	1.68286

Figure S2. Typical raw GC-MS-SIM chromatogram of high (A: PBAT-1) and low (B: PBAT-2) concentration samples (12.37 min, DEA; 13.77, 2, 6-DIPA; 16.77, TBA)

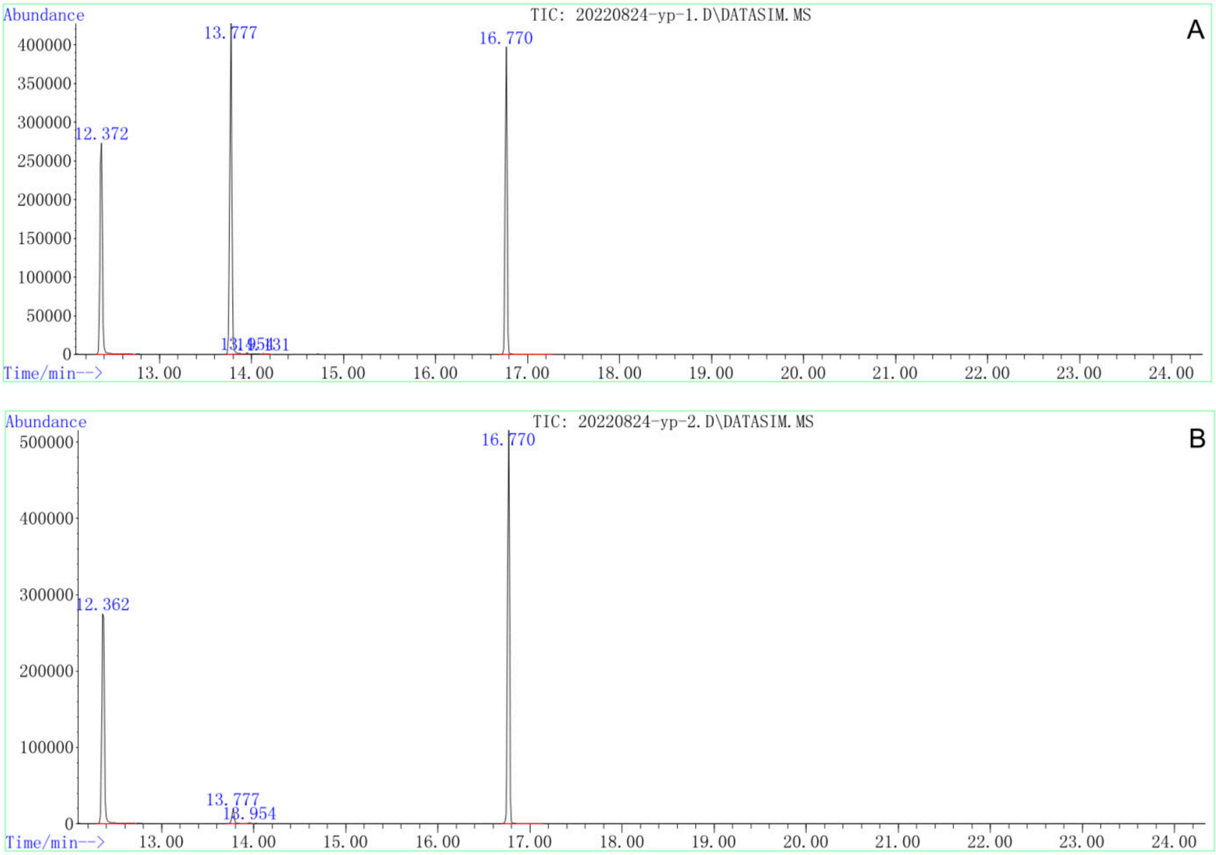

Supplement: Supplementary file 1 [file molecules-29-02068-s001.zip › molecules-2944663-SI.pdf]
